# Comparative Study of Microstructure, Phase Composition, Hardness, and Tribological Behavior of Low-Carbon Steel After Electrolytic-Plasma and Laser Surface Hardening

**DOI:** 10.3390/ma19184007

**Published:** 2026-09-20

**Authors:** Laila Sulyubayeva, Dastan Buitkenov, Almasbek Maulit, Balym Alibekova, Sanzhar Bolatov

**Affiliations:** 1 Research Center Surface Engineering and Tribology, Sarsen Amanzholov East Kazakhstan University, Ust-Kamenogorsk 070000, Kazakhstan; lzhurerova@vku.edu.kz (L.S.); dbuitkenov@vku.edu.kz (D.B.); sbolatov@vku.edu.kz (S.B.); 2 PlasmaScience LLP, Oskemen 070018, Kazakhstan; maulit.almas@gmail.com; 3 Research School of Physical and Chemical Sciences, Shakarim University, Semey 071412, Kazakhstan

**Keywords:** electrolytic-plasma hardening, laser hardening, surface hardening, phase composition, α′-martensite, iron oxides, wear resistance

## Abstract

The aim of this study was to comparatively evaluate the effects of electrolytic-plasma surface hardening (EPSH) and laser surface hardening (LSH) on the microstructure, phase state, microhardness, and friction behavior of low-carbon Steel 20. EPSH was performed using a two-stage regime of 320 V for 3 s followed by 200 V for 40 s and a thermocyclic regime of 320 V for 2 s, 200 V for 2 s, and 50 V for 2 s repeated for five cycles. LSH was carried out at a scanning speed of 7 mm/s and 75% of the maximum laser power (approximately 2250 W) using single- and double-pass treatments. The modified layers were characterized by SEM, XRD, microhardness measurements, and dry sliding tribological tests. EPSH produced the strongest hardening response, reaching approximately 600 HV with a hardened-layer depth of 70–100 μm. Double-pass LSH increased the near-surface microhardness to approximately 460 HV. Phase analysis revealed Fe_1−x_O after EPSH, Fe_3_O_4_ after single-pass LSH, and Fe_1−x_O, Fe_3_O_4_, and Fe_2_O_3_ after double-pass LSH. All treatments reduced the coefficient of friction compared with untreated steel. Overall, EPSH provided deeper and stronger hardening, whereas LSH produced more localized surface modification and oxidation.

## 1. Introduction

Low-carbon steels (with a carbon content typically below 0.25%) are among the most widely used structural materials in modern industry. Their widespread use is due to the favorable combination of high ductility, good formability, high resistance to impact loads, and satisfactory weldability at a relatively low production cost [1]. At the same time, their initial ferritic–pearlitic structure generally provides relatively low surface hardness, insufficient wear resistance, and limited resistance to contact-fatigue and corrosion-mechanical failure, resulting in an inadequate service life of such components under friction, abrasive action, and cyclic loading. Recent studies have also demonstrated that the wear behavior of metallic materials is closely associated with their microstructural state and mechanical characteristics, indicating that changes in microstructure and hardness can substantially affect friction and material loss under sliding contact [2]. Consequently, their use without additional treatment under severe operating conditions characterized by high friction, abrasive wear, or intense contact fatigue is highly limited [3,4,5].

Improving the mechanical strength and wear resistance of low-carbon steels is a major metallurgical challenge [6]. Recent tribological studies have shown that wear behavior is governed by the combined effects of microstructural state, surface hardness, contact conditions, and the mechanisms of material degradation. In particular, microstructural modification and strengthening can significantly influence friction and material loss during sliding contact, whereas deterioration of surface integrity and a decrease in hardness can accelerate wear [7]. The main difficulty is associated with their inherently low carbon content (generally below 0.25 wt.%), which severely limits the through-hardenability of the material during conventional quenching processes [8]. Since a sufficient amount of carbon is required for complete structural transformation into a hard, wear-resistant martensitic phase, direct quenching results in only minimal improvement in properties [9]. Moreover, the use of aggressive rapid-cooling methods in an attempt to intensify hardening can induce severe internal stresses, leading to warping (dimensional distortion) and an increased risk of microcrack formation within the crystalline matrix [10].

To overcome the problem of low through-hardenability of the material, localized surface-hardening methods are commonly applied. Traditional thermochemical treatments, such as pack carburizing, enrich the surface layer with additional carbon to enable martensite formation while leaving the tough, ductile core unaffected [11]. Liquid and gas carburizing variants provide improved thermodynamic control over the carbon diffusion depth and concentration gradients [12]. The resulting hard surface layer significantly improves the wear resistance and impact-load performance of automotive and heavy-engineering components [13]. Nitriding and nitrocarburizing are alternative conventional methods in which nitrogen is introduced to form hard precipitates that modify the microstructural characteristics of the surface layers [14]. However, these conventional processes have significant disadvantages, including long treatment times and a strong dependence on complex high-temperature furnace atmospheres [15]. Even specialized modern modifications, such as low-temperature plasma carburizing, remain limited by stringent vacuum requirements and high energy consumption [16]. In addition, components subjected to conventional electrochemical treatment or carburizing often require subsequent low-temperature tempering or annealing to relieve stresses and reduce brittleness caused by prolonged heating [17].

In contrast, modern high-energy-density methods, particularly laser surface hardening (LSH) and electrolytic-plasma surface hardening (EPSH), offer compelling technological advantages over conventional methods [18]. LSH provides exceptionally precise spatial control, enabling localized non-contact treatment of components with complex geometries without inducing bulk thermal distortion [19]. On the other hand, EPSH operates in environmentally friendly, low-concentration aqueous solutions at atmospheric pressure, eliminating the need for expensive vacuum chambers or hazardous shielding gases [20]. Both methods provide extremely rapid heating and cooling cycles, often completed within seconds rather than hours, enabling the production of near-net-shape components with minimal need for subsequent machining [21].

Numerous previous studies have documented the microstructural and mechanical advantages of these advanced processing methods. LSH studies have shown that focused laser scanning transforms the ferritic–pearlitic matrix of low-carbon steel into fine lath martensite, sharply increasing surface microhardness and tribological performance [22]. It has been demonstrated that applying specialized carbon coatings prior to laser irradiation further improves the phase composition, leading to the formation of cementite–martensite complexes that significantly reduce the coefficient of friction [23]. The rapid self-cooling (self-quenching) inherent in laser processing also refines the grain structure while simultaneously improving both mechanical wear resistance and corrosion resistance [24].

Similarly, extensive studies of EPSH have revealed profound changes in the surface properties of treated steels. For example, the treatment of 40Kh steel resulted in a several-fold increase in microhardness due to the formation of highly dispersed martensitic layers at a precisely optimized holding time [25]. Studies of 30KhGSA steel subjected to thermocyclic electrolytic-plasma treatment revealed the formation of a resilient gradient structure composed of fine martensite transitioning into troostite, which substantially dampens the applied mechanical stresses [26]. Furthermore, controlling the plasma-processing parameters directly governs the surface morphology, sharply reducing the abrasive wear rate through the formation of hard carbide and carbonitride phases [27]. It has been shown that subsequent electropolishing of plasma-hardened layers reduces surface roughness and further optimizes the tribological profile of the steel [28]. In the specific case of low-carbon 20 steel, plasma electrolytic hardening effectively produced dense hardened surface zones, significantly outperforming conventional heating methods in both processing speed and energy efficiency [29]. The application of these principles to agricultural machinery components confirmed that electrolytic-plasma treatment substantially extends the field service life of highly loaded and rapidly wearing components [30]. Moreover, these methods have proven sufficiently versatile for the successful modification of the microstructure and phase states of chromium–nickel alloy steels [31,32].

Although LSH and EPSH have been investigated for several decades, in most published studies these methods are considered separately, with respect to different steels, treatment conditions, and investigated properties. Therefore, a direct comparison of the structural and phase transformations, hardening behavior, and tribological performance produced by two fundamentally different heating methods in the same low-carbon steel remains relevant. Such a comparison makes it possible to determine how differences in the mechanisms of local energy input and subsequent cooling affect the formation and properties of the hardened surface layer.

The aim of this work is to establish the relationship between the energy parameters of localized heating, the formation of the phase composition and morphology of the hardened layer, and the mechanical and tribological properties of low-carbon steel after laser and electrolytic plasma surface hardening under identical experimental conditions. The comparison is carried out using the same initial steel and a unified set of structural, phase, mechanical, and tribological analysis methods.

## 2. Materials and Methods

### 2.1. Materials and Sample Preparation

For electrolytic-plasma surface hardening, structural low-carbon Steel 20 was selected as the material under investigation; its international equivalent is AISI 1020 steel. Steel 20 belongs to the group of low-carbon structural steels used for manufacturing machine parts and structural components that require a combination of ductility, weldability, and moderate strength. In terms of chemical composition, it is comparable to AISI 1020 steel; however, these grades should not be considered completely identical because the allowable ranges of the elements are regulated by different standards. The chemical composition of grade 20 steel according to GOST 1050-88 [33] is presented in Table 1. The test samples were made in the form of rectangular parallelepipeds measuring 20 × 20 × 15 mm. Before electrolytic plasma treatment, the sample surfaces were subjected to successive mechanical grinding to achieve a uniform surface with a metallic luster. After grinding, the samples were thoroughly rinsed with running water to remove abrasive particles, machining residue, and other surface contaminants. The prepared samples were dried and used directly for subsequent electrolytic plasma surface hardening.

### 2.2. Electrolytic-Plasma Surface Hardening

**Figure 1:**
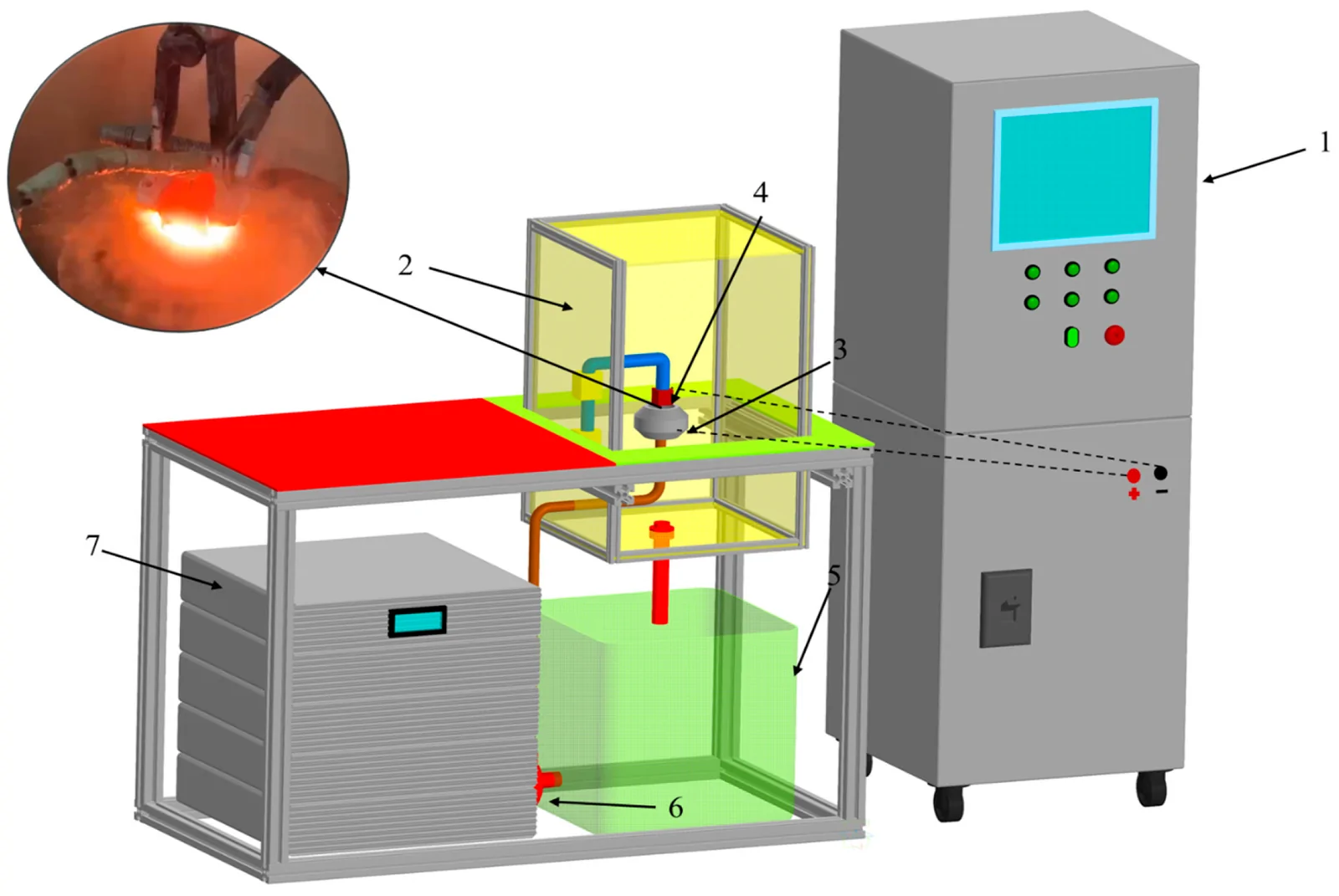
Electrolytic-Plasma Material Processing System [34].

Electrolytic plasma surface hardening (EPSH) of the investigated samples was carried out using a specialized installation for electrolytic plasma treatment of materials, the schematic diagram of which is shown in Figure 1. The installation was developed and manufactured at the Research Center “Surface Engineering and Tribology”. The treatment process was carried out according to the following technological scheme. The working electrolyte contained in the bath (5) was supplied by a chemically resistant pump (6) to the plasma torch (3) installed inside the working chamber (2). After passing through the treatment zone, the electrolyte was discharged through the drain holes and returned to the bath, thereby ensuring its continuous circulation in a closed loop. The working fluid flow rate was maintained within the range of 4–7 L/min, which ensured the stable formation of a vapor–gas envelope and sustained electrical discharge in the near-surface region of the sample. To prevent electrolyte overheating and maintain constant temperature conditions, a heat exchanger (7) was used to stabilize the working medium temperature within the range of 40–50 °C.

The sample (4) was fixed in the working chamber so that the surface to be treated was positioned opposite the plasma torch nozzle outlet at a distance of 2–3 mm. A jet of electrolyte was directed through the nozzle onto the sample surface, ensuring continuous renewal of the working medium in the treatment zone, efficient heat removal, and subsequent intensive cooling of the heated surface layer. Maintaining a constant interelectrode distance contributed to the stable formation of the vapor–gas envelope and the stable operation of the electrolytic plasma discharge. The electrical connection of the installation was implemented according to the cathodic configuration. The anode was connected to the positive terminal of the power supply (1), while the treated sample, acting as the cathode, was connected to the negative terminal. When the operating voltage was applied between the electrolytic anode and the metallic cathode, an electrical discharge occurred, providing rapid heating of the sample surface to the phase transformation temperature. After the voltage was switched off, intensive cooling by the electrolyte flow promoted hardening of the material’s surface layer. Two types of electrolytic plasma surface hardening were carried out: two-stage hardening, in which a high voltage of 320 V was initially applied for 3 s to ignite the arc, after which the plasma was maintained at a voltage of 200 V for 40 s, and thermocyclic hardening, in which a high voltage of 320 V was applied for 2 s to ignite the arc, after which the plasma was maintained at a voltage of 200 V for 2 s and cooled at 50 V for 2 s. This procedure was repeated for 5 cycles. In all treatments, the sample was cooled in the same electrolyte.

### 2.3. Laser Surface Hardening

**Figure 2:**
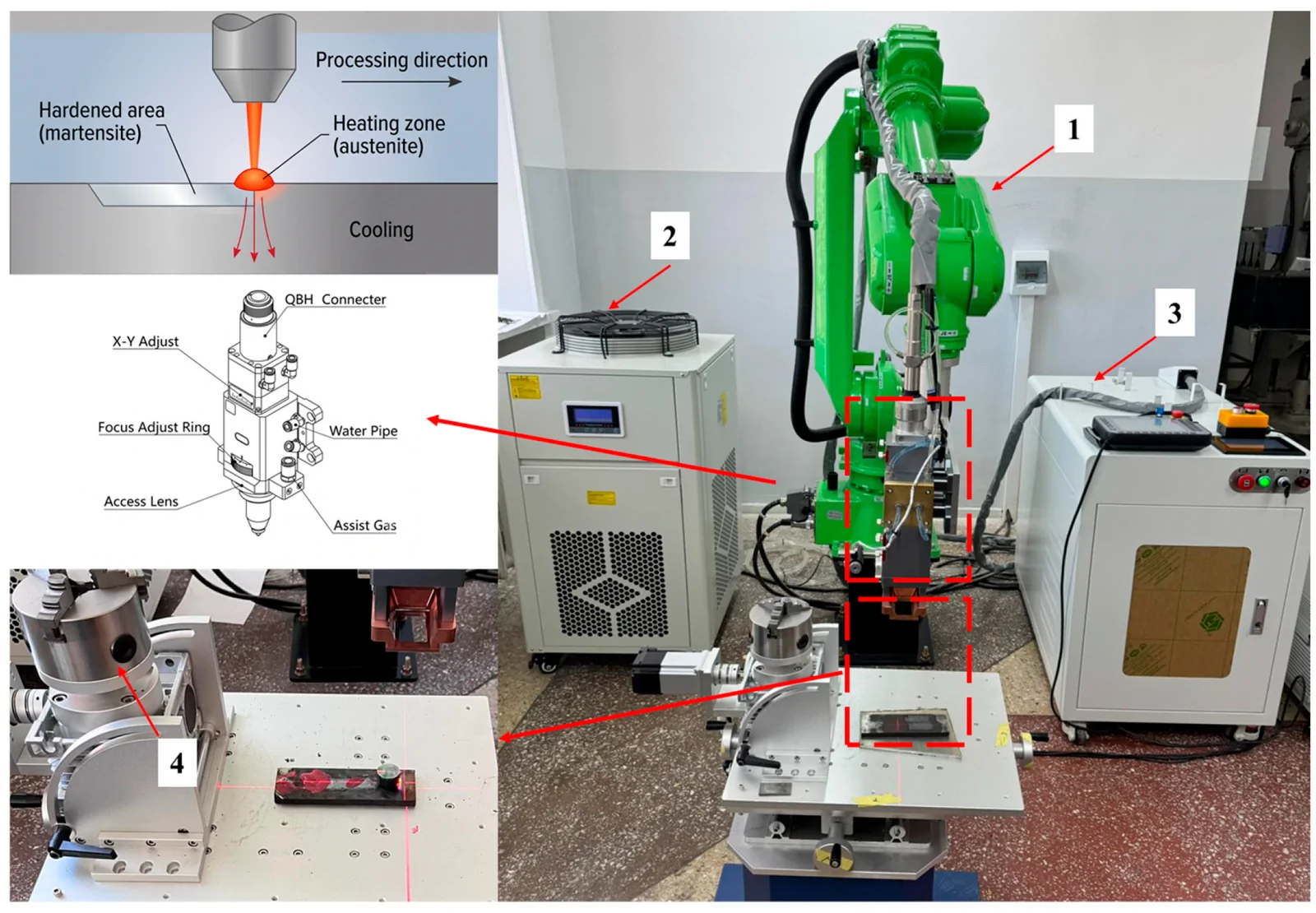
Installation for laser hardening of materials.

Laser hardening was carried out using a diode (semiconductor) laser with a power of 3000 W, a frequency of up to 5 kHz, and a wavelength of 915–976 nm (Figure 2). The samples were mounted in a special holder (4), scanning was performed using a robot (1), the hardening time and power were set via the power supply (3), and the plasma torch was cooled using a chiller (2). Single-pass and double-pass laser hardening were carried out at a scanning speed of 7 mm/s and 75% (2250 W) of the maximum power. In all treatments, the sample was cooled in air at room temperature. The electrolytic plasma surface hardening conditions are presented in Table 2.

Laser treatment was carried out in an air atmosphere without the use of a shielding gas. Therefore, interaction of the heated surface with oxygen from the air was possible during treatment, and the formation of surface oxides was subsequently taken into account when interpreting the XRD results. After the laser beam passed, cooling occurred mainly due to heat transfer from the locally heated surface region into the colder bulk of the material and heat exchange with the surrounding environment.

### 2.4. Investigation of Microstructure and Phase Composition

X-ray phase analysis of the investigated samples was performed using a DX-27mini benchtop X-ray diffractometer manufactured by Dandong Haoyuan Instrument Co., Ltd. (Dandong, China). The instrument was equipped with an X-ray tube with a copper anode operating at an accelerating voltage of 40 kV and a current of 30 mA. The diffraction patterns were recorded using Cu-Kα radiation with a wavelength of λ = 1.541 Å. The measurements were performed over a diffraction angle range of 2θ from 30° to 90°, with a scanning step of 0.02° and a signal acquisition time of 0.5 s at each point. The obtained diffractograms were used to identify the crystalline phases present, evaluate changes in the phase composition, and analyze the structural and phase transformations occurring in the material as a result of surface treatment.

The microstructure and local chemical composition of the samples were investigated using a SEM3200 scanning electron microscope (CIQTEK Co., Ltd., Hefei, China) equipped with an energy-dispersive X-ray microanalysis system. Prior to microscopic examination, the prepared metallographic specimens were etched in a 4% nitric acid solution, which enabled the grain boundaries, structural constituents, and transition zones of the treated layer to be revealed. Microstructural analysis made it possible to evaluate the morphology of the surface and cross-section, the degree of uniformity of the formed layer, and the presence of defects, pores, microcracks, and other characteristic structural features. Energy-dispersive analysis was used to determine the elemental composition of individual surface regions and to evaluate the distribution of chemical elements through the thickness of the modified layer.

### 2.5. Microhardness Measurement

The mechanical characteristics of the material were evaluated by instrumented indentation using a FISCHERSCOPE HM2000 microhardness tester manufactured by Helmut Fischer GmbH (Sindelfingen, Germany). The measurements were performed at a maximum load of 100 mN. Indentations were made sequentially at several points on the investigated surface or along the depth of the sample cross-section. To improve the reliability of the results, a series of measurements was performed in each investigated region, after which the average microhardness values were calculated. The obtained data were used to evaluate the degree of surface-layer hardening, the uniformity of hardness distribution, and the effective depth of the applied treatment. The obtained “microhardness–depth” profiles were used to evaluate the maximum hardening effect, the hardness gradient, and the approximate depth of the mechanically modified region, rather than only the maximum surface hardness value.

### 2.6. Tribological Testing

**Figure 3:**
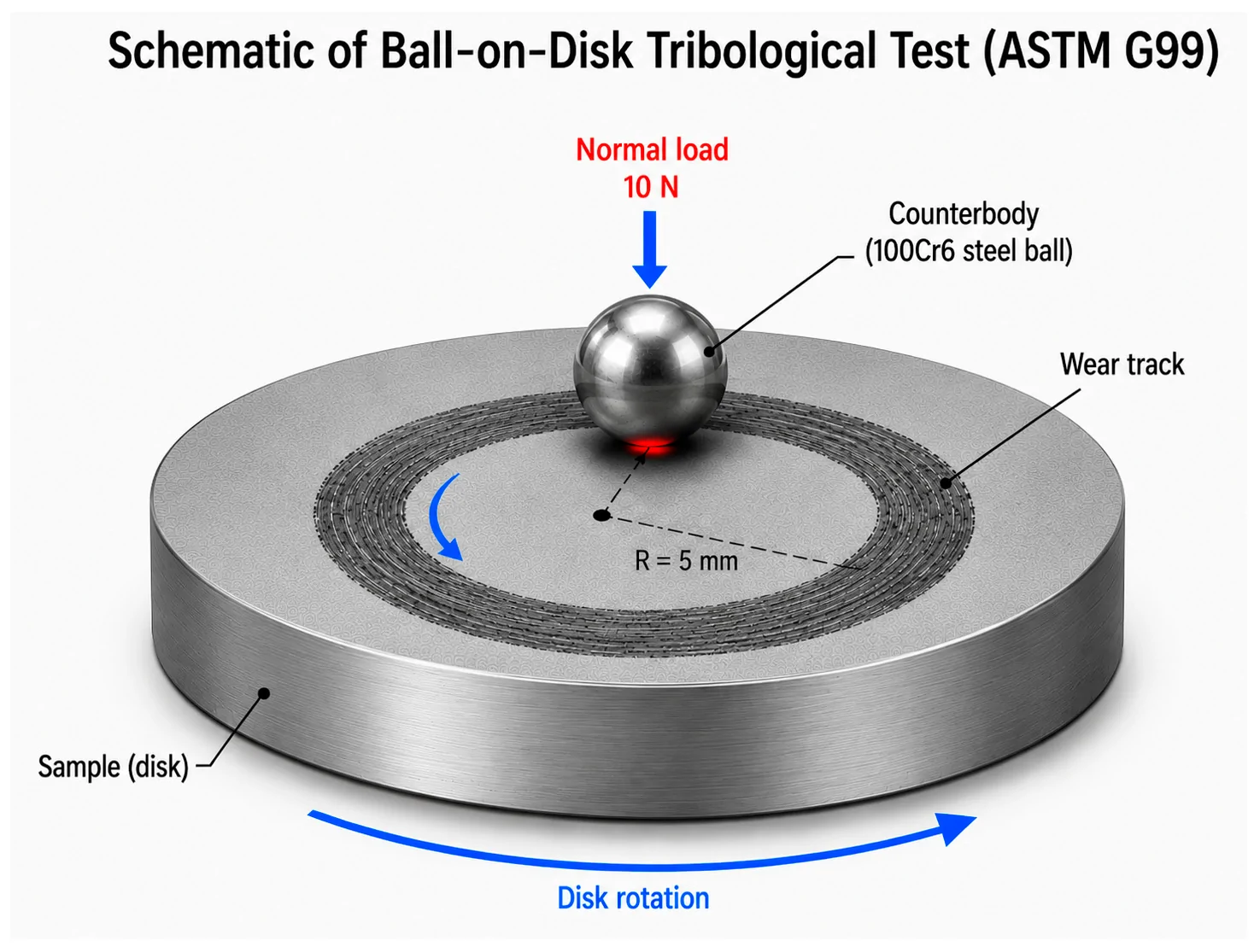
Schematic representation of the ball-on-disk tribological test in accordance with ASTM G99.

The tribological characteristics of the samples were investigated using a TRB^3^ universal tribometer manufactured by Anton Paar (Graz, Austria). The tests were conducted using the ball-on-disk configuration in accordance with the requirements of ASTM G99 [35]. The experiments were performed under dry-friction conditions, without lubricants, at an ambient temperature of 25 ± 1 °C. A ball made of 100Cr6 bearing steel was used as a stationary counterbody and was pressed against the surface of the investigated sample under a vertical load of 10 N. The relative sliding speed was 0.05 m/s, and the total sliding distance was 100 m (Figure 3).

The principle of the ball-on-disk test consisted in creating localized contact between the spherical counterbody and the flat surface of the sample. The investigated sample was secured on the rotating stage of the tribometer, whereas the steel ball was fixed in a holder and pressed against the surface under a specified normal load. As the sample rotated, the ball moved relative to its surface along a circular trajectory, resulting in the formation of an annular wear track. The tangential force arising in the contact zone was continuously recorded by the sensitive sensor of the instrument. The coefficient of friction was determined as the ratio of the measured friction force to the applied normal load.

## 3. Results and Discussions

### 3.1. Investigation of Microstructure and Phase Composition

**Figure 4:**
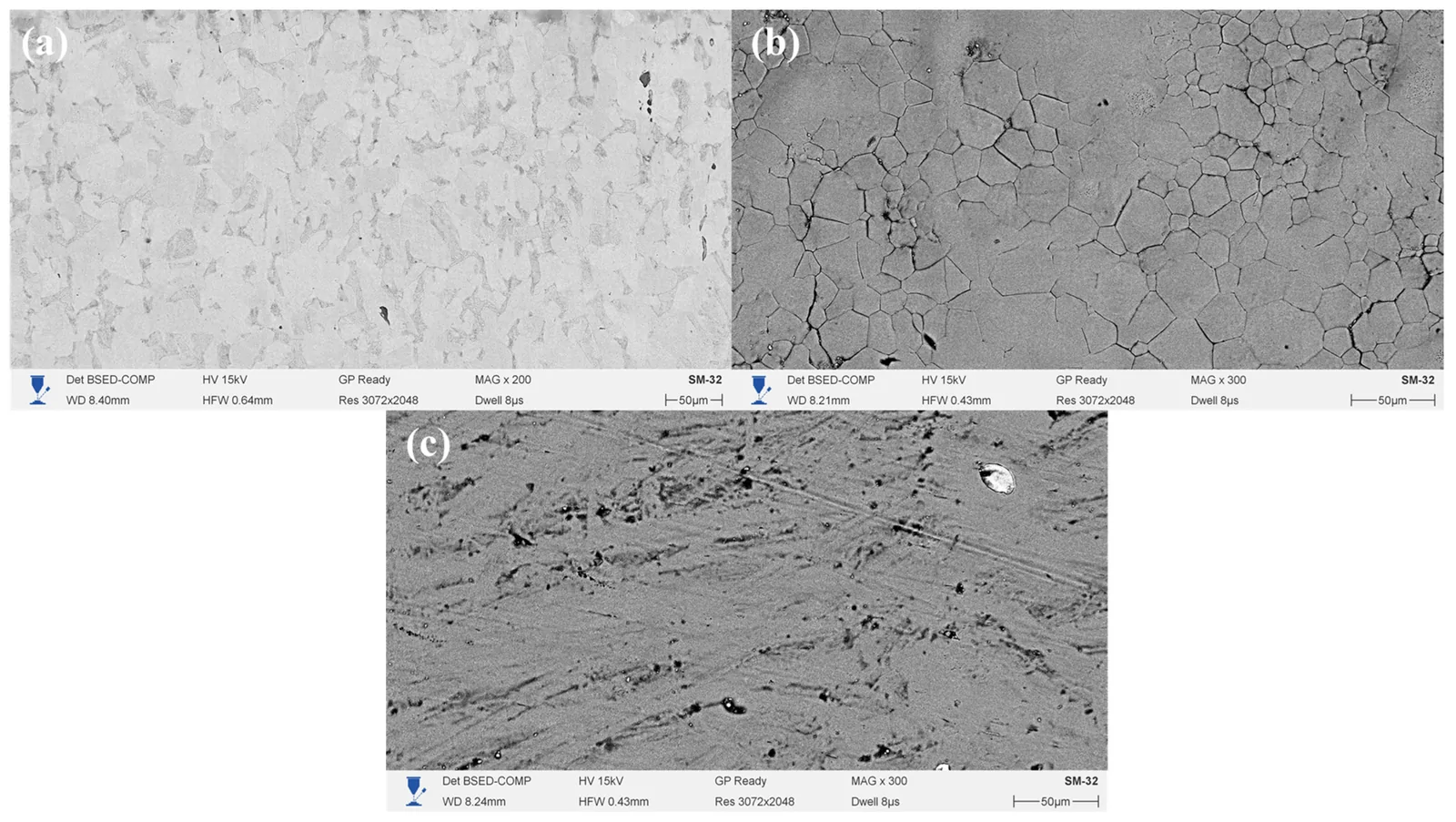
SEM images of the surface of Steel 20 in the initial and hardened conditions: (**a**) initial condition; (**b**) after electrolytic-plasma hardening at 320 V–2 s and 200 V–40 s; (**c**) after single-pass laser hardening at a scanning speed of 7 mm/s and a power of 75% of the maximum.

Figure 4 presents SEM images of the surface of Steel 20 in the initial condition and after two types of surface hardening. The initial surface (Figure 4a) is characterized by a comparatively homogeneous ferrite–pearlite microstructure typical of low-carbon steel, without pronounced signs of intensive thermal exposure. After electrolytic-plasma hardening (Figure 4b), the surface morphology changes significantly: a pronounced grain-boundary relief is formed, areas with different degrees of etching are observed, and local surface discontinuities are present. Such changes may be associated with rapid surface heating, phase transformation during austenitization, subsequent intensive cooling, as well as interaction of the heated metal with the electrolytic-plasma environment. After laser hardening (Figure 4c), the surface retains a smoother character; at the same time, directional relief features, local dark defects, and traces of non-uniform modification associated with the movement of the localized laser-heating zone are observed. Unlike electrolytic-plasma treatment, laser exposure does not lead to such a pronounced restructuring of the surface relief.

The obtained SEM data are in good agreement with the surface roughness measurements. For the first specimen after electrolytic-plasma hardening at 320 V–2 s and 200 V–40 s, the Ra parameter increased from 0.18 to 1.12 μm, i.e., by approximately 6.3 times. An even more pronounced change was observed after thermocyclic electrolytic-plasma treatment at 320 V–2 s, 200 V–2 s, and 50 V–2 s for 5 cycles: Ra increased from 0.12 to 2.98 μm, corresponding to an increase of approximately 24.5 times. The significant increase in roughness after electrolytic-plasma treatment indicates intensive modification of the surface layer. Probable contributing factors include localized high-rate heating, cyclic thermal expansion and contraction, formation of oxide products, as well as local surface erosion under conditions of interaction between the plasma envelope and the metal. The particularly high Ra value after five cycles indicates the cumulative nature of the surface changes during repeated heating and cooling.

Laser hardening has a significantly smaller effect on surface roughness. After single-pass treatment at a scanning speed of 7 mm/s and a power of 75%, Ra changed from 0.12 to 0.17 μm, corresponding to an increase of approximately 36.8%. After two passes under the same parameters, the roughness increased from 0.17 to 0.21 μm, which corresponds to an increase of approximately 23.7%. The slight change in Ra indicates that, under the selected parameters, laser hardening predominantly provides thermal modification of the near-surface layer without significantly disturbing the initial surface geometry. At the same time, the repeated pass does not cause a sharp increase in roughness, despite the more pronounced phase and oxidation changes previously revealed by X-ray diffraction.

**Figure 5:**
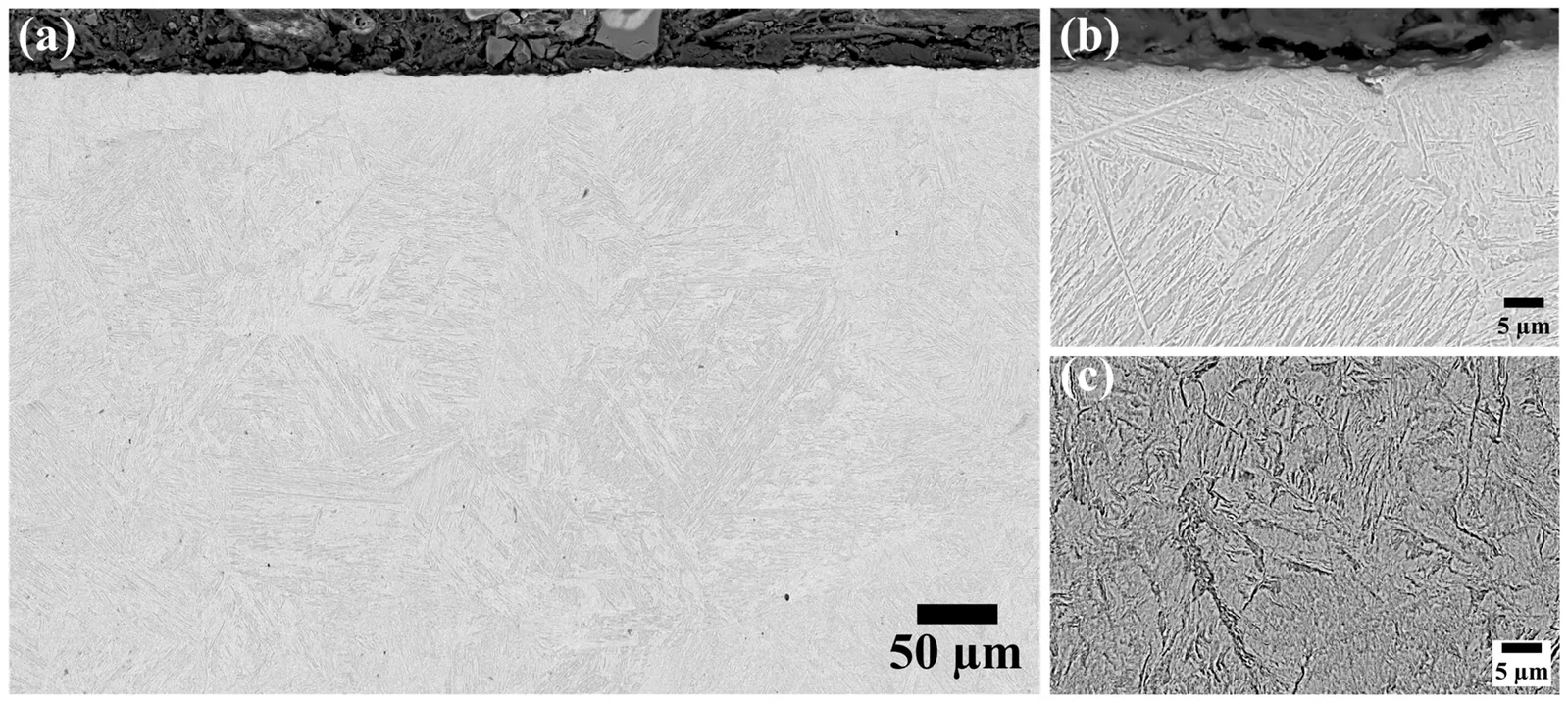
SEM images of the cross-section of steel 20 after electrolytic plasma hardening at 320 V for 2 s and 200 V for 40 s: (**a**) overview of the modified layer, ×200; (**b**) microstructure of the near-surface zone, ×2000; (**c**) microstructure of the inner region of the hardened layer, ×2000.

In the SEM image in Figure 5 of the cross-section of sample 1, treated by electrolytic plasma hardening at 320 V for 2 s and 200 V for 40 s, the formation of a structurally modified near-surface zone is observed. In the overview image at ×200 magnification (Figure 5a), the metallic matrix remains continuous; no extended cracks or large internal discontinuities were detected in the examined region. At the interface with the external environment, a morphologically heterogeneous layer with a developed relief and local defects is present. Considering the X-ray phase analysis results, this layer may be associated with the formation of Fe_1−x_O; however, its phase identification based solely on BSE contrast is insufficient and requires local SEM/EDS analysis. A distinct boundary between the hardened zone and the initial metallic matrix is not observed at low magnification, indicating a gradual change in the structure with depth.

At ×2000 magnification in the near-surface zone (Figure 5b), packets of thin laths and plates are revealed, predominantly parallel within individual regions and intersecting at different angles between adjacent packets. Such needle-like lath morphology, together with the α-Fe/α′ reflections and increased microhardness values, allows this region to be interpreted as a structure predominantly consisting of low-carbon lath martensite. Similar formation of a fine lath-needle martensitic structure after electrolytic plasma heating and rapid cooling has been established for low-carbon steel 20 and low-alloy structural steels [36,37,38]. These studies showed that short-term high-rate heating ensures surface austenitization, while subsequent cooling due to heat dissipation into the bulk metal and contact with the electrolyte leads to martensitic transformation.

In the deeper region of the modified layer (Figure 5c), the lath-plate morphology is retained; however, coarsening of individual structural elements, reduced prominence of parallel packets, and increased heterogeneity of the internal relief are observed. This indicates the formation of a gradient structure caused by a decrease in the maximum temperature and cooling rate with increasing distance from the surface. In this region, martensitic and untransformed ferritic constituents may coexist; however, their quantitative differentiation based on the presented SEM image is not possible. A gradient change in structure and hardness with depth was previously established during electrolytic plasma hardening of low-carbon steel 20, with the onset of martensitic transformation being associated with reaching a critical cooling rate of approximately 50 °C/s [32]. A similar evolution from a heterogeneous, partially transformed structure to a dense lath martensitic morphology with increasing electrolytic plasma treatment duration was also observed for steel 45 [39].

**Figure 6:**
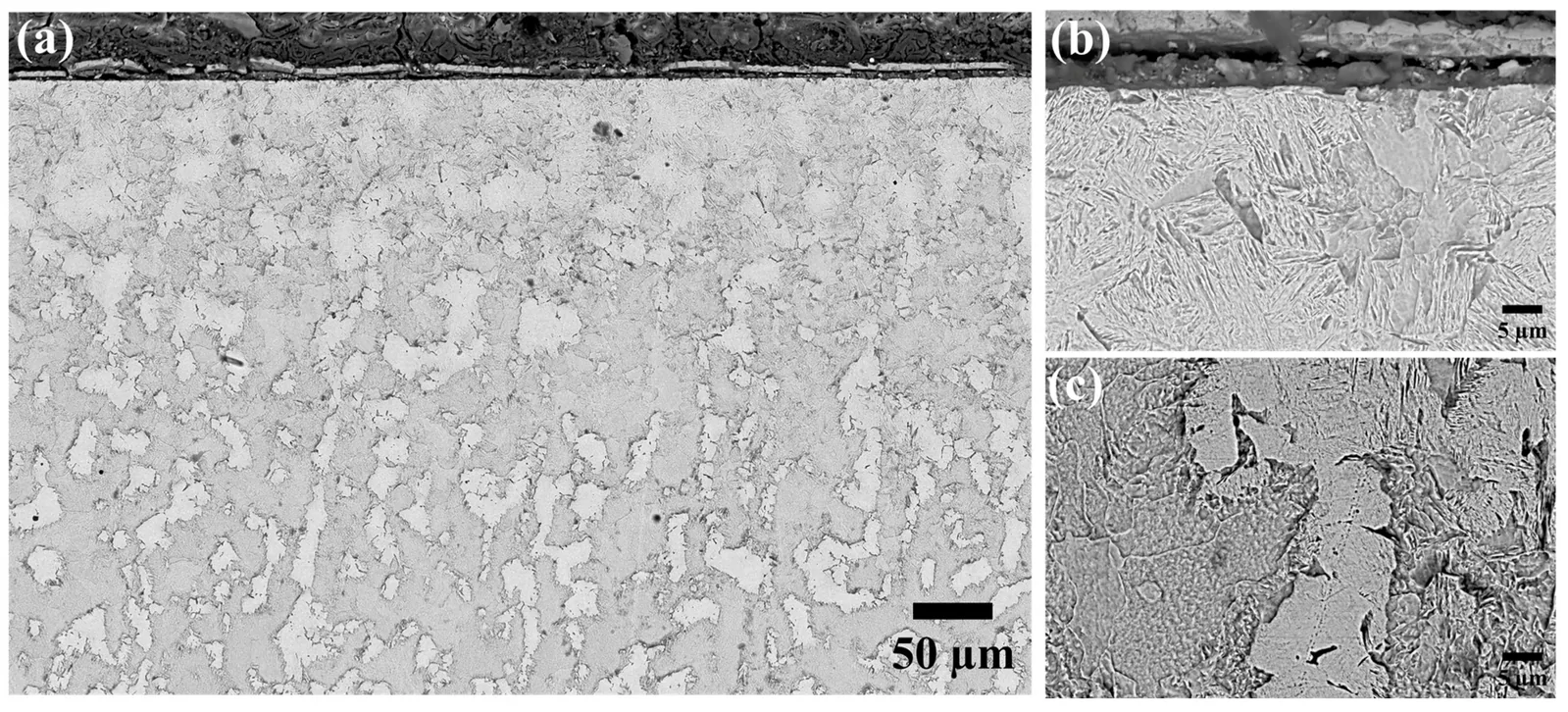
SEM images of the cross-section of steel 20 after two-pass laser hardening at a speed of 7 mm/s and 75% power: (**a**) overview of the modified zone and surface oxide layer, ×200; (**b**) lath-plate structure of the near-surface zone, ×2000; (**c**) structural heterogeneity of the inner region of the modified layer, ×2000.

In the SEM image in Figure 6 of the cross-section of steel 20 after two-pass laser hardening at a beam travel speed of 7 mm/s and 75% power, pronounced structural heterogeneity of the modified zone is revealed. In the overview image at ×200 magnification (Figure 6a), alternating light regions of irregular polygonal shape and darker dispersed areas are observed. A heterogeneous layer of variable thickness with local discontinuities and a developed relief is formed along the surface. Similar formation of a structurally modified layer in AISI 1020 low-carbon steel after laser heating is attributed to short-term austenitization followed by self-cooling of the material due to intensive heat dissipation into the cold core of the sample [40].

At ×2000 magnification in the near-surface region (Figure 6b), packets of thin laths and plates with different spatial orientations are observed, forming a characteristic needle-like lath morphology. Considering the XRD results, this structure can be attributed predominantly to low-carbon α′-martensite together with regions of retained α-phase. The formation of a martensitic constituent after laser treatment of AISI 1020 was previously confirmed by comparing the microstructure, thermal cycles, and hardness of the treated zone [23]. At the same time, the image contains relatively large regions with weakly developed internal relief, indicating incomplete uniformity of the phase transformation due to the spatial distribution of absorbed laser energy and cooling rate.

In the image of the inner part of the modified zone (Figure 6c), a combination of large polygonal regions and surrounding dispersed lath-plate areas is observed, along with local dark discontinuities near the boundaries of the structural constituents. The repeated laser pass is accompanied by reheating of the previously hardened zone; therefore, reaustenitization, additional martensitic transformation, or local tempering of the previously formed martensite may occur in individual regions. Similar heterogeneity of the two-pass laser-hardened zone and the formation of regions of retransformation and tempering-induced softening have been described for low-alloy structural steel subjected to two-track laser treatment [41]. The surface layer with pronounced heterogeneity is also consistent with data on laser treatment of AISI 1020 in air, where interaction of the heated surface with oxygen leads to oxidation; the use of inert argon, in contrast, reduces this effect [42].

**Figure 7:**
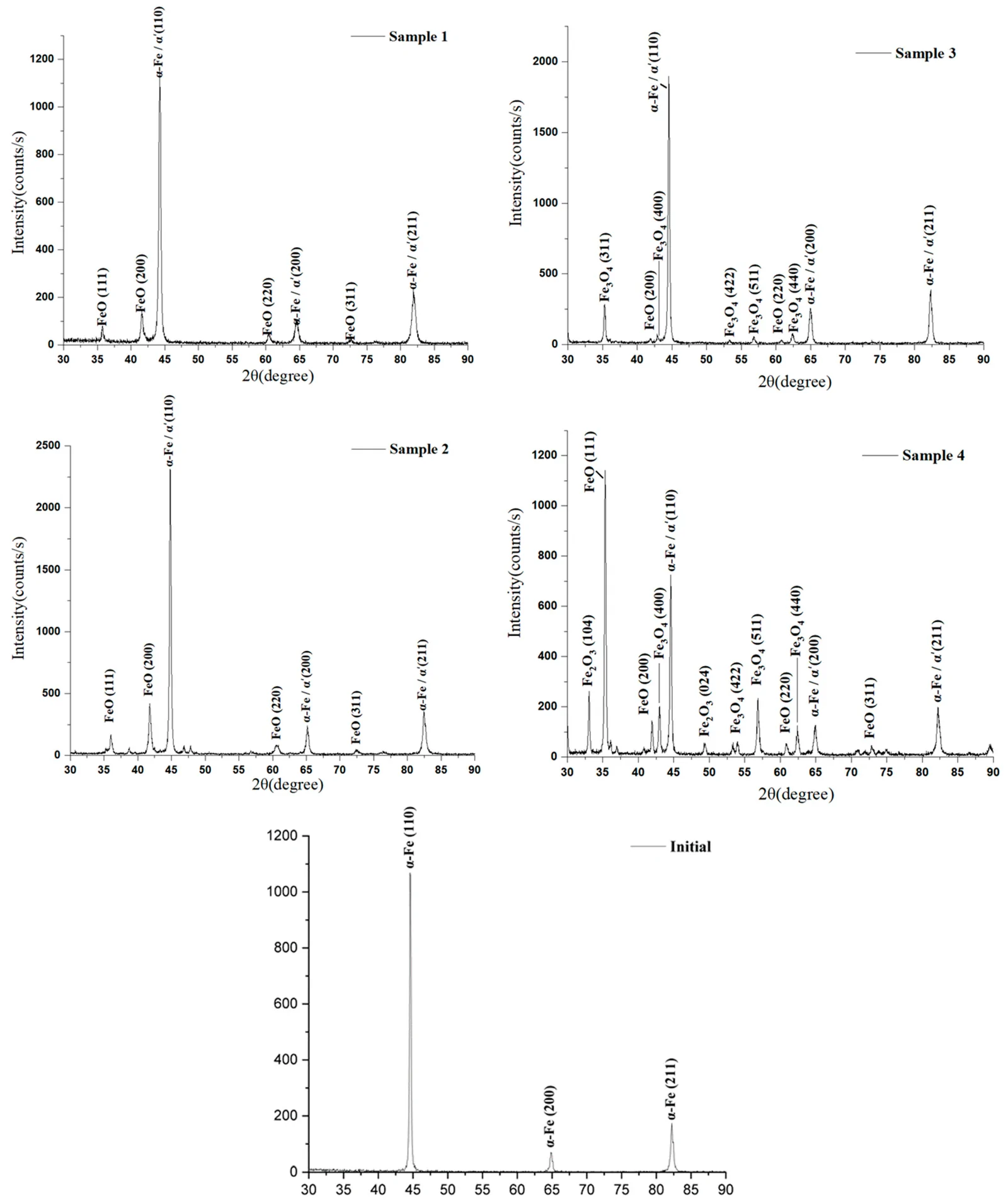
X-ray diffraction patterns of the initial steel 20 and the samples after electrolytic plasma hardening (samples 1 and 2) and laser hardening (samples 3 and 4).

As shown in Figure 7, the diffraction pattern of the initial sample is characterized by α-Fe reflections at 2θ ≈ 44.7°, 65.0°, and 82.3°, corresponding to the (110), (200), and (211) planes of the BCC lattice. After all treatment regimes, these metallic reflections were retained and were assigned to α-Fe/low-carbon α′-martensite. The differentiation of ferrite and martensite based on the presented diffraction patterns is difficult due to the low tetragonality of martensite in low-carbon steel.

In samples 1 and 2, additional Fe_1−x_O reflections were identified, corresponding predominantly to the (111), (200), (220), and (311) planes. This indicates the formation of a wüstite-like oxide layer during electrolytic plasma heating and subsequent rapid cooling. The phase composition of the samples after continuous holding for 30 and 40 s does not differ fundamentally: in both cases, the metallic constituent is represented by α-Fe/α′, while the main additional phase is Fe_1−x_O. The thermocyclic regime also does not lead to the formation of new metallic phases; however, it is accompanied by more pronounced oxide reflections, which may be associated with the repeated heating and cooling cycles.

After laser hardening, a more complex oxide-layer composition is observed. In sample 4, along with the α-Fe/α′ reflections, magnetite Fe_3_O_4_ lines were identified, including the (311), (400), (422), (511), and (440) reflections, as well as weak Fe_1−x_O reflections. After two laser passes, the intensity of the oxide peaks in sample 5 increases significantly, and hematite Fe_2_O_3_ reflections additionally appear. Consequently, repeated laser exposure leads to the development of a multiphase oxide layer containing Fe_1−x_O, Fe_3_O_4_, and Fe_2_O_3_. At the same time, a consistent series of retained austenite γ-Fe reflections was not detected in any of the investigated samples. Since the intensity scales of the individual diffraction patterns differ, comparison of the phase contents based on the absolute peak heights should be regarded as qualitative; quantitative assessment requires profile normalization and full-pattern analysis.

### 3.2. Investigation of Mechanical Properties

**Figure 8:**
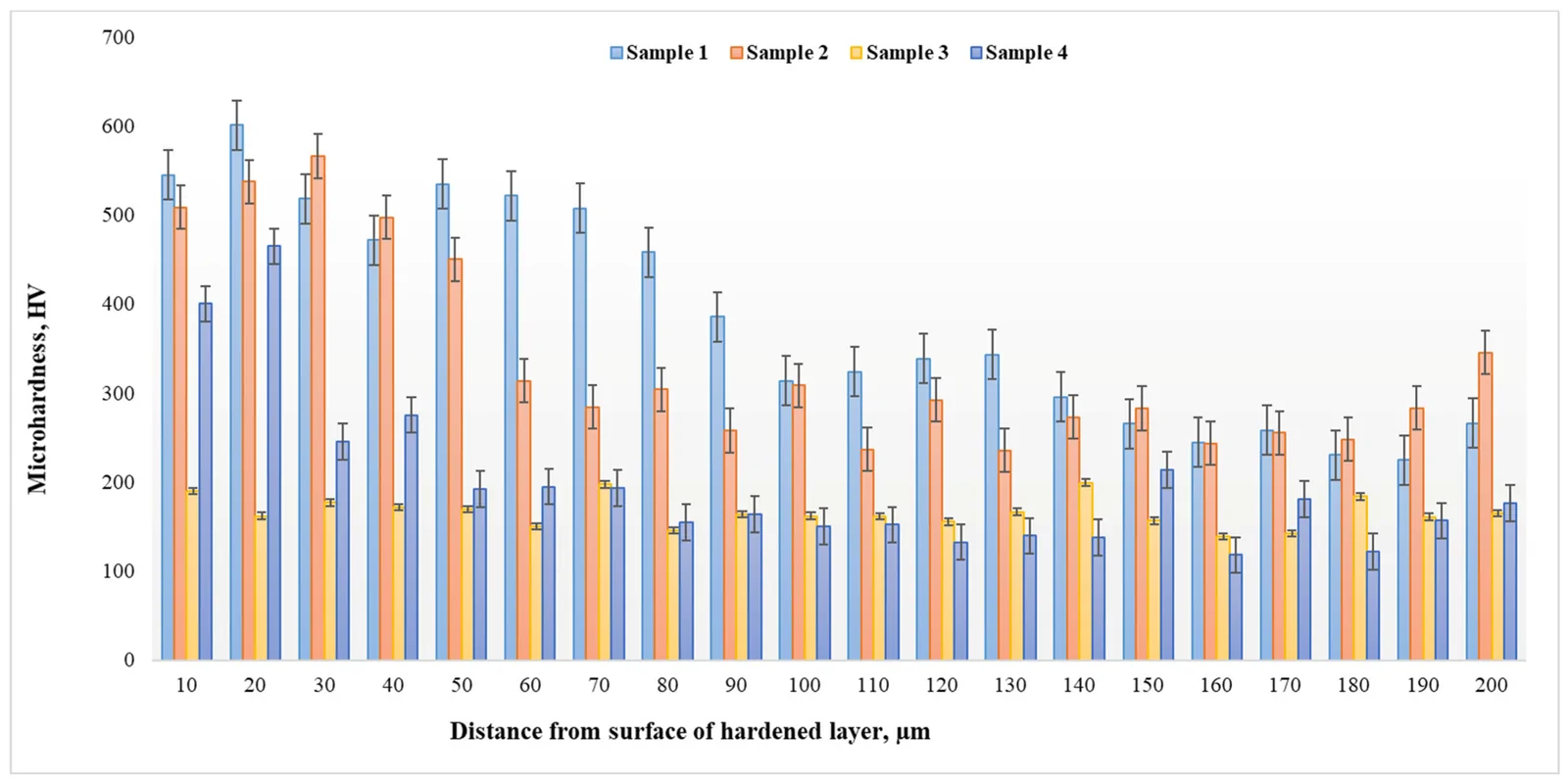
Microhardness profiles of steel 20 as a function of the distance from the treated surface after electrolytic plasma hardening (samples 1 and 2) and laser hardening (samples 3 and 4).

Figure 8 presents the microhardness profiles of steel 20 as a function of the distance from the treated surface in the range of 10–200 µm. Sample 1, after electrolytic plasma hardening at 320 V for 2 s and 200 V for 40 s, is characterized by the highest and most extensive hardening. In the near-surface zone, the microhardness is approximately 530 HV and reaches a maximum of about 600 HV at a depth of 20 µm. In the range of 30–70 µm, the values remain at 470–530 HV, after which they gradually decrease to 300–340 HV at a depth of 100–130 µm and to 220–270 HV in the 150–200 µm region. The obtained profile indicates the formation of a relatively deep structurally modified layer with a gradual transition to the less hardened inner region. Sample 2, after thermocyclic electrolytic plasma hardening, is also characterized by high microhardness near the surface: the values increase from approximately 510 HV at a depth of 10 µm to 565 HV at a depth of 30 µm. However, beyond 50–60 µm, a more pronounced decrease in microhardness to 280–310 HV is observed, indicating a smaller effective hardening depth compared with Sample 1.

Sample 3, after single-pass laser hardening, is characterized by the most uniform but comparatively low microhardness profile—approximately 135–195 HV throughout the investigated depth. The absence of a pronounced near-surface maximum indicates the limited effectiveness of this hardening regime, which may be associated with insufficient absorbed energy density, a shallow modified zone, or the predominant contribution of the initial ferrite–pearlite structure to the measurement results.

For Sample 4, after two-pass laser hardening, pronounced local hardening was observed directly near the surface. The microhardness is approximately 400 HV at a depth of 10 µm and reaches 460 HV at a depth of 20 µm, after which it sharply decreases to 245–275 HV in the 30–40 µm range and to 190–200 HV at a depth of approximately 50–70 µm. In the deeper regions, the values are predominantly within the range of 110–210 HV. Therefore, the repeated laser pass produces a high-hardness but relatively thin surface zone, whereas electrolytic plasma hardening with a holding time of 40 s forms the deepest and most stable hardening gradient. Nonlinear fluctuations in the individual profiles and the magnitude of the confidence intervals reflect local structural heterogeneity caused by different proportions of martensitic, ferritic, and oxide constituents, as well as by indentations falling within the boundary regions of structural elements. Based on the overall results, Sample 1 exhibits the most effective regime in terms of the simultaneously achieved microhardness and hardening depth, whereas Sample 4 demonstrates the greatest effect of laser treatment only within a narrow near-surface zone.

**Figure 9:**
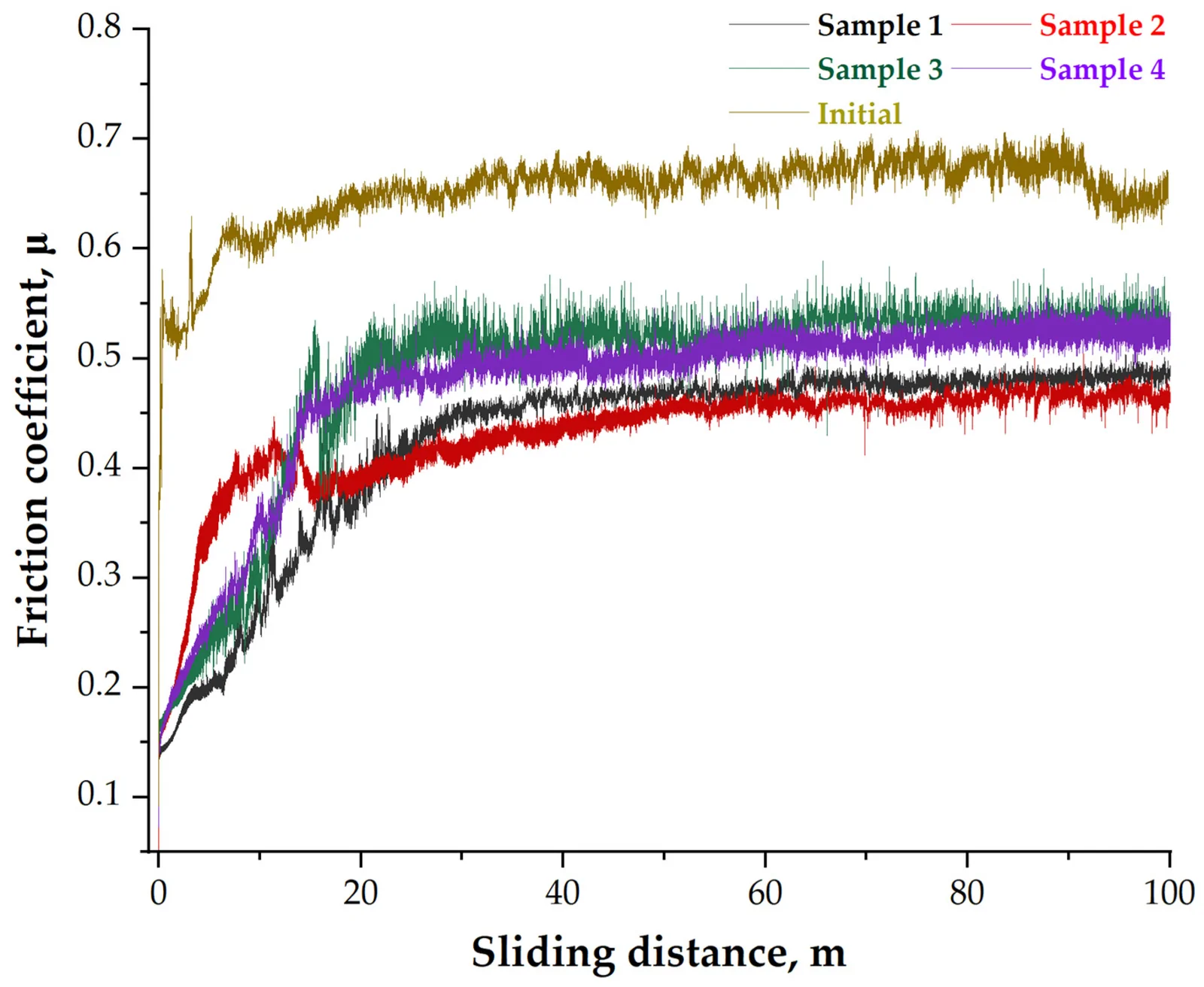
Dependence of the coefficient of friction of steel 20 on the sliding distance for the untreated sample, after electrolytic plasma hardening (Samples 1 and 2), and after laser hardening (Samples 3 and 4).

Figure 9 presents the dependence of the coefficient of friction of steel 20 on the sliding distance in the initial state and after five surface-hardening regimes. For all treated samples, a running-in stage is observed in the initial test region, characterized by an increase in the coefficient of friction from μ ≈ 0.14–0.16 to a quasi-steady-state level. The running-in period is approximately 15–30 m. On individual curves within this interval, local fluctuations and short-term decreases in the coefficient of friction are recorded, caused by changes in the real contact area, removal of surface asperities, and formation of secondary structures in the tribological contact zone.

The untreated steel is characterized by the highest coefficient of friction. After a short running-in period, its value increases to μ ≈ 0.60–0.65 and reaches approximately 0.66–0.69 in the steady-state regime. For Samples 1–3, after electrolytic plasma treatment, the coefficient of friction decreases significantly. In the steady-state regime, it is approximately 0.47–0.49 for Sample 1, 0.46–0.48 for Sample 2, and 0.44–0.45 for Sample 3. The minimum value was recorded for Sample 3, indicating the most favorable combination of structural state, hardness, and surface-layer stability under friction. The lower coefficient of friction of the treated samples compared with the untreated steel may be associated with the formation of a hardened α-Fe/α′ structure, reduced plastic deformation of the contacting asperities, and the formation of a stable tribological layer.

For Samples 3 and 4 after laser hardening, the steady-state coefficient of friction is approximately 0.53–0.55 and 0.51–0.53, respectively. These values are lower than those of the untreated steel but higher than the coefficients of friction of the samples after electrolytic plasma hardening. The increased amplitude of fluctuations in the curve for Sample 4 may indicate periodic breakdown and reformation of the surface oxide and tribological layers. The second laser pass slightly reduces and stabilizes the coefficient of friction; however, the developed multiphase oxide scale containing Fe_1−x_O, Fe_3_O_4_, and Fe_2_O_3_ does not provide the same low level of friction as the electrolytic plasma treatment of Sample 3. Thus, all the investigated regimes reduce the coefficient of friction compared with the untreated steel, with the regime used for Sample 3 being the most effective.

The frictional behavior correlates with the structural and mechanical changes induced by surface hardening. After EPSH, the highest microhardness (approximately 565–600 HV) and the lowest coefficient of friction (0.45–0.49) were achieved, whereas the laser-treated specimens were characterized by lower hardness and intermediate coefficient of friction values (0.50–0.55). The obtained results show that the frictional behavior is determined by the combined influence of surface hardening, structural changes, and oxidation. However, since the wear volume and wear rate were not determined, a lower coefficient of friction should not be directly interpreted as higher wear resistance.

**Figure 10:**
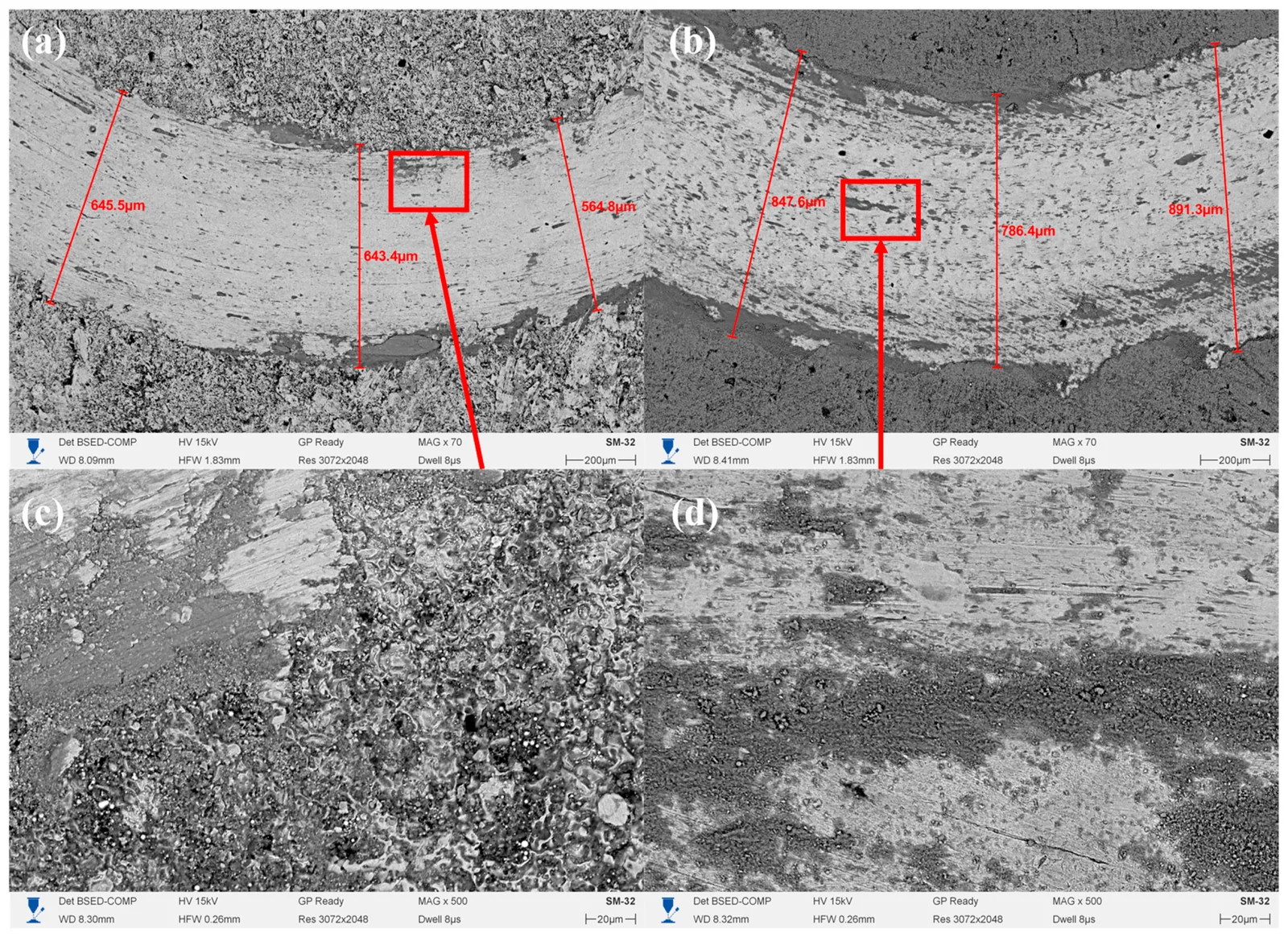
SEM images of the wear tracks of Steel 20 after tribological testing: (**a**) after electrolytic-plasma hardening at 320 V–2 s and 200 V–40 s; (**b**) after double-pass laser hardening at a scanning speed of 7 mm/s and a power of 75% of the maximum; (**c**,**d**) enlarged images of characteristic areas of the wear tracks of specimens (**a**) and (**b**), respectively.

Figure 10 presents SEM images of the wear tracks of Steel 20 after tribological testing: (Figure 10a,c) after electrolytic-plasma hardening at 320 V–2 s and 200 V–40 s; (Figure 10b,d) after double-pass laser hardening at a scanning speed of 7 mm/s and a power of 75% of the maximum. For the electrolytic-plasma-hardened specimen, the width of the wear track varies approximately from 564.8 to 645.5 μm, with an average value of about 618 μm. The enlarged image (Figure 10c) shows longitudinal grooves, local areas of plastic deformation, and accumulations of wear debris; at the same time, most of the track surface retains a relatively compact structure without developed regions of large-scale damage. Such morphology indicates a combination of abrasive wear with local adhesive and oxidative processes. After double-pass laser hardening (Figure 10b), the wear track is significantly wider, measuring approximately 786.4–891.3 μm, with an average value of about 842 μm. The enlarged image (Figure 10d) demonstrates more pronounced longitudinal sliding marks, non-uniformly distributed areas of plastic deformation, local delamination, and accumulation of wear debris. Compared with the electrolytic-plasma-treated specimen, the track after laser hardening is approximately 36% wider, indicating more intensive destruction of the surface layer under the investigated friction conditions. The observed morphology is consistent with the previously established tribological characteristics: electrolytic-plasma hardening provides a more stable surface condition during sliding, whereas after double-pass laser treatment, the more pronounced structural and oxide heterogeneity promotes local damage and an increase in the size of the wear zone.

**Figure 11:**
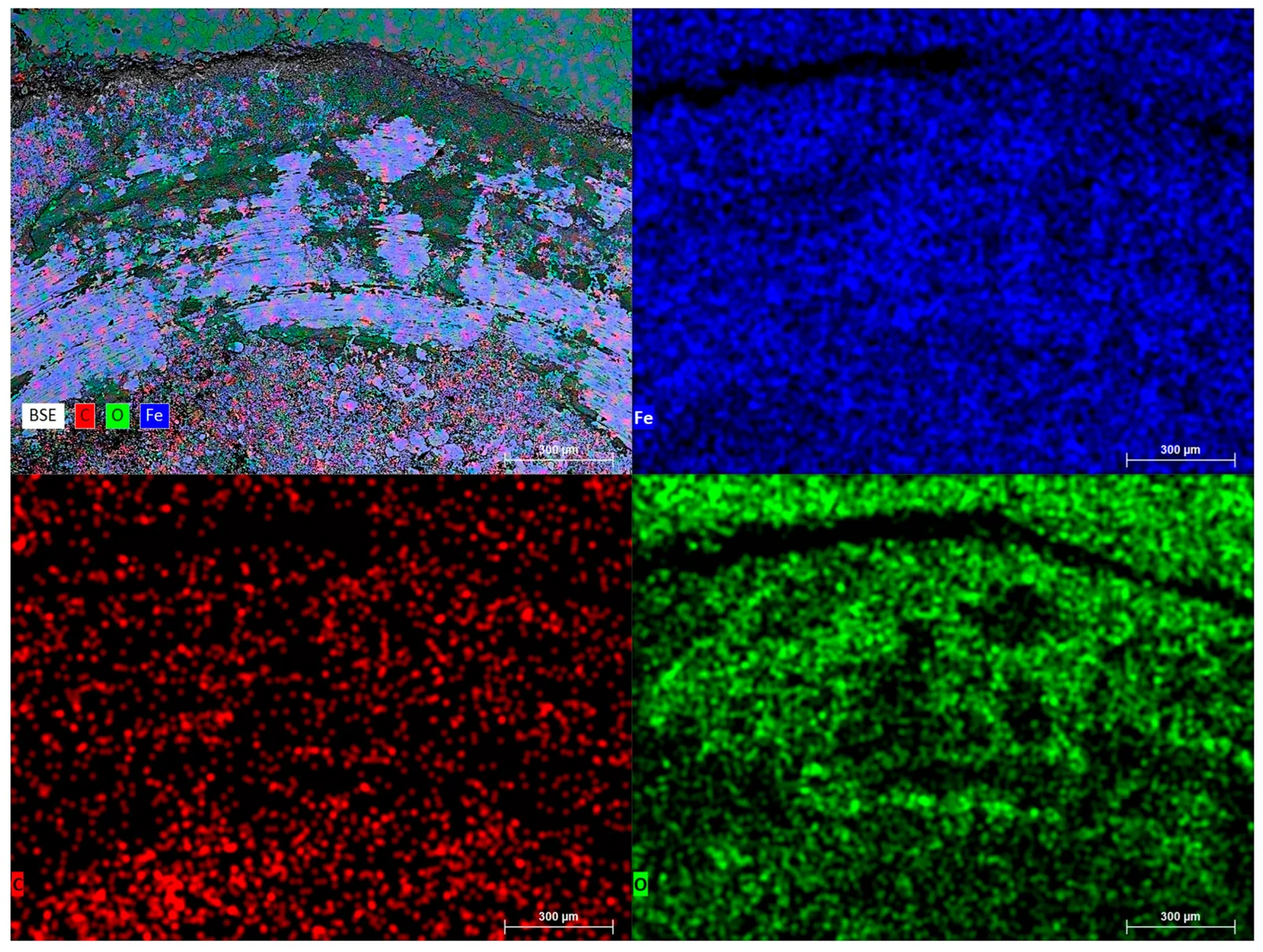
EDS mapping of the distribution of Fe, C, and O in the wear-track region of Steel 20 after electrolytic-plasma hardening at 320 V–2 s and 200 V–40 s and subsequent tribological testing.

EDS mapping of the wear track of Steel 20 (Figure 11) after electrolytic-plasma hardening at 320 V–2 s and 200 V–40 s revealed a spatially non-uniform distribution of Fe, O, and C. Iron is the dominant element and is distributed over most of the investigated surface; however, in certain areas of the wear track, a decrease in the Fe signal intensity is observed, coinciding with regions of increased oxygen content. The O distribution map demonstrates pronounced local oxygen enrichment, particularly in morphologically altered regions and areas containing accumulated wear debris, indicating the occurrence of tribo-oxidative processes and the formation of an oxygen-containing secondary layer during sliding. Considering the previously established presence of Fe_1−x_O after electrolytic-plasma treatment by XRD, the increased oxygen concentration in the wear track may be associated both with the preservation of initially formed oxide products and with the additional formation of iron oxides directly during tribological contact. Carbon is distributed discretely in the form of locally enriched regions; however, due to the limited accuracy of EDS in the analysis of light elements and the possible contribution of surface contamination, its distribution does not allow an independent carbon-containing phase to be identified unambiguously. Overall, the EDS mapping results confirm that wear of electrolytic-plasma-hardened Steel 20 is accompanied by a combination of mechanical interaction and local tribo-oxidation, with the formation and redistribution of secondary wear products on the wear-track surface.

**Figure 12:**
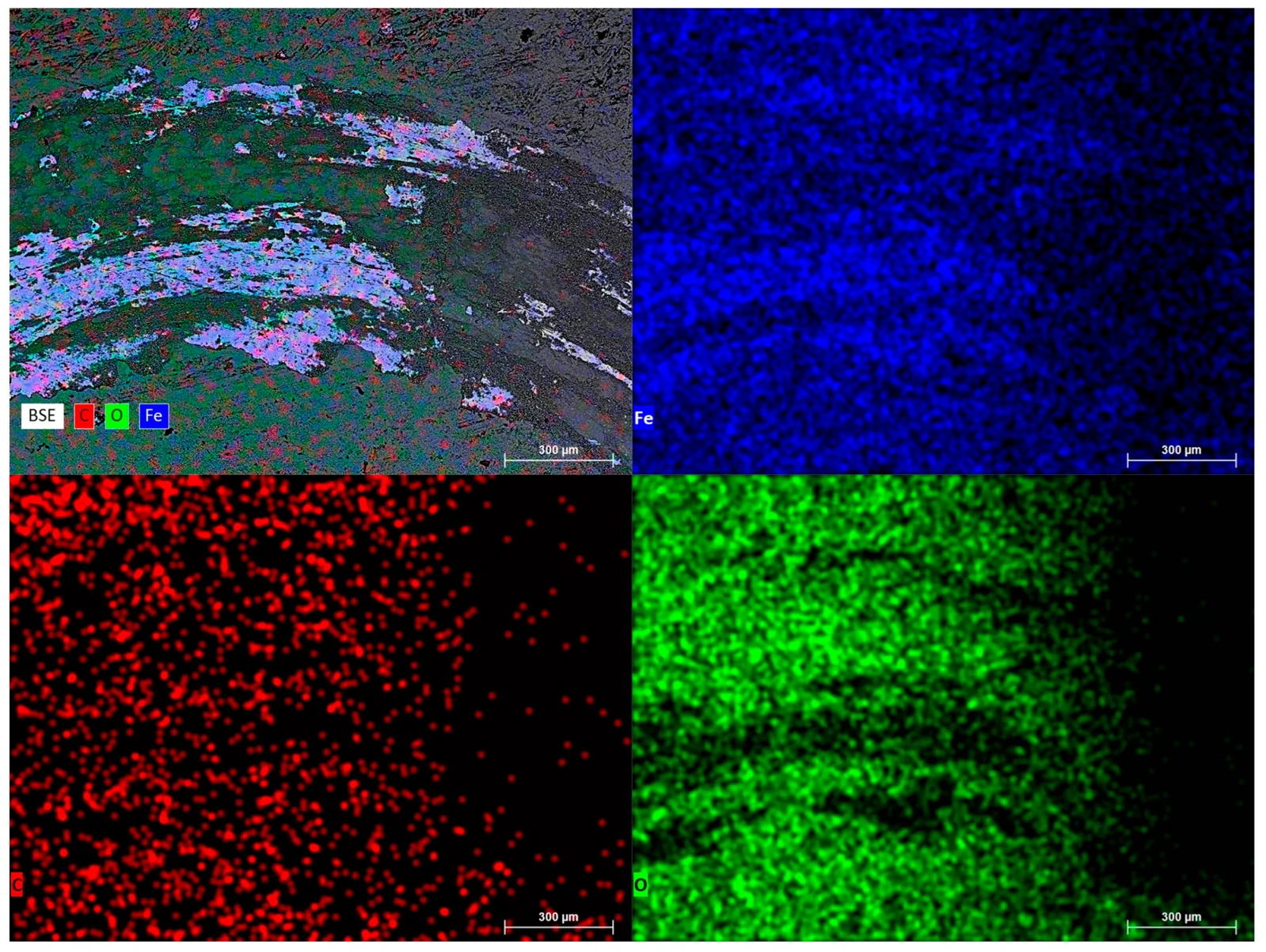
EDS mapping of the distribution of Fe, O, and C in the wear-track region of Steel 20 after double-pass laser hardening at a scanning speed of 7 mm/s and a power of 75% of the maximum and subsequent tribological testing.

EDS mapping of the wear track of Steel 20 (Figure 12) after double-pass laser hardening at a scanning speed of 7 mm/s and a power of 75% of the maximum revealed pronounced chemical heterogeneity of the surface after tribological testing. Iron is distributed over the main part of the investigated area, while its intensity varies locally in accordance with the morphology of the wear track. The oxygen map shows significant O enrichment in individual regions, with areas of increased oxygen signal spatially coinciding with zones of altered contrast and accumulation of wear debris in the BSE image. The co-localization of Fe and O indicates the formation of iron oxide products during friction and the development of a tribo-oxidative wear mechanism. This is consistent with the X-ray diffraction results, according to which Fe_1−x_O, Fe_3_O_4_, and Fe_2_O_3_ are present in the surface layer after double-pass laser treatment; however, EDS mapping does not allow the individual oxide phases to be distinguished. The distribution of carbon is discrete and highly non-uniform; therefore, its local enrichment may be associated with products of tribological interaction and surface contamination and cannot be regarded as unambiguous evidence of the formation of an independent carbon-containing phase. Overall, the obtained data confirm intensive local oxidation of the wear track after double-pass laser hardening and the formation of a heterogeneous secondary tribological layer involved in the surface wear process.

Figure 10, Figure 11 and Figure 12 also provide evidence of abrasive and adhesive wear mechanisms. The scratches and grooves observed within the wear tracks indicate a contribution from abrasive wear, whereas localized regions of material transfer suggest the occurrence of adhesive wear. These observations indicate that the overall wear process involves a combination of tribo-oxidative, abrasive, and adhesive wear mechanisms rather than a single dominant mechanism.

## 4. Conclusions

The comparative study demonstrated that electrolytic-plasma surface hardening (EPSH) and laser surface hardening (LSH) produce distinctly different structural and mechanical responses in low-carbon Steel 20 as a consequence of their different heating and cooling mechanisms.

EPSH provided the strongest hardening response, with a maximum microhardness of approximately 600 HV and a hardened depth of about 70–100 μm for the two-stage regime of 320 V for 3 s followed by 200 V for 40 s. Laser surface hardening produced a more localized modification; however, repeated laser exposure increased the near-surface microhardness to approximately 460 HV.

The treatments also resulted in different oxidation behavior. EPSH was mainly associated with Fe_1−x_O formation, whereas laser processing in ambient air promoted Fe_3_O_4_ formation and, after repeated exposure, a more complex oxide composition containing Fe_1−x_O, Fe_3_O_4_, and Fe_2_O_3_.

Both surface-hardening approaches reduced the coefficient of friction compared with untreated Steel 20. The greater reduction observed after EPSH corresponded to its more pronounced hardening response, whereas the laser-treated samples showed intermediate friction values.

Overall, EPSH produced deeper and stronger surface hardening, while LSH provided a more localized modification of the surface region. The comparison demonstrates how differences in localized energy input and subsequent cooling govern the resulting microstructure, hardness, oxidation behavior, and friction response of low-carbon Steel 20.

## Figures and Tables

**Table 1:** Chemical composition of Steel 20.

Grade	C	Si	Cr	Mn	Ni	Cu	P	S	As
Steel 20	0.17–0.24	0.17–0.37	up to 0.24	0.35–0.65	up to 0.3	up to 0.25	up to 0.035	up to 0.04	up to 0.08

**Table 2:** Electrolytic plasma surface hardening conditions.

Samples	Type of Hardening	Hardening Conditions	Medium	Pass
Sample 1	EPSH	320 V—3 s	Water-based electrolyte 12% Na_2_CO_3_ Distilled water 88%	1
		200 V—40 s		
Sample 2	EPSH	320 V—2 s		5
		200 V—2 s		
		50 V—2 s		
Sample 3	Laser	7 mm/s,	Air	1
Sample 4	Laser	75% power		2

## Data Availability

The original contributions presented in this study are included in the article. Further inquiries can be directed to the corresponding author.
